# CHARACTERIZING SYMPTOM BURDEN AMONG COMMUNITY-DWELLING OLDER ADULTS

**DOI:** 10.1093/geroni/igad104.3219

**Published:** 2023-12-21

**Authors:** Michelle McKay, Paul Bernhardt, Melissa O’Connor, Suzanne Leveille

**Affiliations:** Villanova University, Villanova, Pennsylvania, United States; Villanova University, Villanova, Pennsylvania, United States; Villanova University, Villanova, Pennsylvania, United States; UMass Boston, Boston, Massachusetts, United States

## Abstract

One-third of symptoms reported by older adults are unexplained and not directly related to a chronic disease diagnosis. This ambiguity contributes to undertreatment and chronic functional burdens in vulnerable older patients. Previous studies have found that older adults often experience multiple co-occurring symptoms that contribute to disablement. Symptom burden in older adults increases healthcare utilization, decrease physical performance, and leads to poorer quality of life. This study aimed to characterize symptom burden in a population-based cohort of community-dwelling older adults enrolled in the MOBILIZE Boston study (n=765). We used descriptive statistics for symptom prevalence including pain, balance, weakness, endurance, sleep difficulty, depression/anxiety and sensory impairments. We used latent class analysis to identify 4 distinct classes based on overall symptom burden: 1) mild (26.6%), 2) moderate (53.8%), 3) moderate-severe (12%) and 4) severe (7.5%). Moderate to high levels of pain, the most prevalent symptom, were reported in all symptom burden classes except the mild class. Older adults with severe symptom burden, group 4, experienced worse levels of all symptoms compared to other classes. They also experienced moderate endurance and weakness symptoms, and mild and/or severe balance symptoms. Older adults in the moderate-severe class tended to also report mild endurance, balance, and weakness symptoms. Understanding overall symptom burden, both in terms of numbers and severity of symptoms, is the first step in determining the impact of symptom burden as a possible new clinical indicator for fall risk and other detrimental health outcomes.

